# Primary Low-Grade Endometrial Stromal Sarcoma of the Ovary Arising From Endometriosis: A Case Report

**DOI:** 10.7759/cureus.79530

**Published:** 2025-02-23

**Authors:** Yuri Tenjimbayashi, Yusuke Kobayashi, Yurina Suzuki, Toyomi Satoh, Koji Horie

**Affiliations:** 1 Department of Obstetrics and Gynecology, Institute of Medicine, University of Tsukuba, Tsukuba, JPN; 2 Department of Gynecology, Saitama Cancer Center, Saitama, JPN

**Keywords:** estrogen receptor, low-grade endometrial stromal sarcoma, medroxyprogesterone acetate, ovarian endometriosis, ovarian tumor, progesterone receptor

## Abstract

Low-grade endometrial stromal sarcoma (LGESS) is a rare mesenchymal tumor of female genital tract malignancies. While it primarily arises in the uterus, extrauterine cases, including those originating in the ovary, are exceedingly rare. While there is a hypothesis that endometriosis plays a role in the development of extrauterine cases, the diagnosis of primary ovarian LGESS is challenging due to its rarity and similarity to metastatic uterine LGESS.

A 52-year-old premenopausal woman with a history of adenomyosis was referred for an enlarging left ovarian endometriotic cyst, raising suspicion of malignancy. Transvaginal ultrasound revealed a 100×50 mm left ovarian mass with a 30 mm thick cystic component. MRI indicated a solid-cystic tumor with features suggestive of an endometriotic cyst. Positron emission tomography-computed tomography (PET-CT) revealed mild fluorodeoxyglucose (FDG) uptake with a maximum standardized uptake value (SUVmax) of 3.05, with no evidence of distant metastases. Primary debulking surgery was performed. Intraoperatively, the left ovary was enlarged (10 cm) with peritoneal dissemination. A frozen section suggested a granulosa cell tumor. The patient underwent total hysterectomy, bilateral salpingo-oophorectomy, subtotal omentectomy, and high anterior rectal resection. Histopathology revealed a proliferation of small, uniform tumor cells resembling proliferative endometrial stroma adjacent to an area of ovarian endometriosis. Immunohistochemical analysis showed positivity for estrogen receptor (ER), progesterone receptor (PgR), and CD10, confirming LGESS. The final diagnosis was the International Federation of Gynecology and Obstetrics (FIGO) Stage IIIB (pT3bNxM0). Postoperatively, the patient was treated with medroxyprogesterone acetate (MPA) at 600 mg/day, later reduced to 400 mg/day due to weight gain. She remains recurrence-free three years post-treatment.

Primary ovarian LGESS is rare and often difficult to distinguish from metastatic uterine LGESS. The presence of endometriosis suggests a link to chronic inflammation and estrogen stimulation in tumorigenesis. Strong ER/PgR positivity highlights its endocrine-dependent nature, making hormonal therapy an effective option. MRI findings, including high T1 signal, low T2 signal, and heterogeneous enhancement, may aid in diagnosis, especially when combined with endometriosis. Optimal cytoreductive surgery improves prognosis, and complete resection of disseminated lesions likely contributed to this patient’s favorable outcome. Hormonal therapy plays a crucial role in preventing recurrence, though long-term management must consider adverse effects.

This case highlights the importance of considering LGESS in ovarian tumors associated with endometriosis. A combination of optimal surgery and hormonal therapy is key to long-term remission. Close monitoring of ovarian endometriosis patients is essential due to the risk of malignant transformation.

## Introduction

Endometrial stromal sarcoma (ESS) is a rare mesenchymal tumor that accounts for approximately 0.2% of malignant tumors of the female genital tract [1], usually arising from the endometrium but rarely from extrauterine sites such as the ovary or vagina [2]. According to the WHO 2020 classification, ESS is classified into four types which are endometrial stromal nodule (ESN), low-grade endometrial stromal sarcoma (LGESS), high-grade endometrial stromal sarcoma (HGESS), and undifferentiated uterine sarcoma (UUS). LGESS is a tumor that usually originates from the endometrial stroma of the uterus and has a relatively good prognosis. Primary ovarian LGESS can also occur, but are extremely rare and their etiology and pathogenesis are not fully understood. From a small number of case series, the age of onset ranged from 34 to 61 years (median 51.5 years), and the diagnosis was usually triggered by abdominal findings such as abdominal distention and abdominal pain (71.4%), but 21.4% were asymptomatic and discovered incidentally [3]. Tumors were mostly unilateral (78.6%), 4-18 cm in size (mean 9.5 cm), and solid or solid-cystic type [3]. Pathologically, small, uniform tumor cells resembling proliferative endometrial stroma grow in a spiral pattern around small vessels, with round or oval nuclei, sparse cytoplasm, and are characterized by CD10, estrogen receptor (ER), and progesterone receptor (PgR) positivity [3]. Hormonal therapy in addition to surgery is considered effective and a relatively good prognosis has been reported with a recurrence rate of 33.3% [3].

In addition, there are anecdotal reports of LGESS occurring in the setting of ovarian endometriotic cysts [2,4] suggesting that endometriosis may play an important role in tumorigenesis. In addition, LGESS is often ER and PgR positive, making hormone therapy a treatment option, and establishing a diagnosis and treatment strategy is challenging [3].

In this report, we present a rare case of LGESS arising from an ovarian endometriotic cyst that underwent optimal surgery and hormonal therapy with a good postoperative course.

## Case presentation

**Figure 1:**
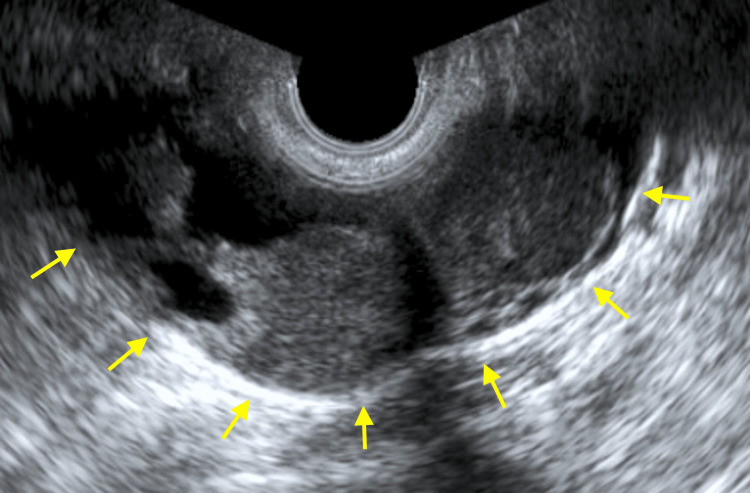
Ultrasound findings. Enlarged left ovary and internal solid component observed on transvaginal ultrasound.

The patient was 52 years old, had experienced three pregnancies and two deliveries, and was in a premenopausal phase. Her medical and family histories did not reveal any significant features. Five years earlier, she had been diagnosed with uterine adenomyosis at another hospital, and she had been under their care since then. She was referred to our hospital because of an enlarged ovarian endometriotic cyst on transvaginal ultrasound, and malignancy could not be ruled out. At the initial examination, the uterus was the size of a goose egg, and a cyst, comparable in size to a newborn’s head, was palpable in the left adnexal region with poor mobility. Transvaginal ultrasound showed that the left ovary was enlarged to 100x50 mm with a 30 mm thick cyst inside (Figure 1).

Blood analysis showed no significant abnormalities in hemogram and biochemistry, while tumor markers showed a high level of CA125 at 99.8 U/ml (reference ranges <40) and a slightly high level of squamous cell carcinoma antigen at 1.6 ng/mL (reference ranges <1.5) (Table 1).

**Table 1:** Laboratory investigations. Abnormal values and their items are in bold. WBC: white blood cells; RBC: red blood cells; AST: aspartate aminotransferase; ALT: alanine aminotransferase; LDH: lactate dehydrogenase; BUN: blood urea nitrogen; CRP: C-reactive protein; APTT: activated partial thromboplastin time; PT: prothrombin time; CEA: carcinoembryonic antigen; SCC: squamous cell carcinoma antigen; HE4: human epididymis protein 4; ul: microliter; g/dl: grams per deciliter; mg/dl: milligrams per deciliter; U/l: units per liter; mmol/l: millimole per liter; µg/ml: micrograms per milliliter; U/ml: units per milliliter; ng/ml: nanograms per milliliter; pmol/l: picomole per liter

Laboratory investigation (unit)	Before surgery	Reference range
WBC (10^3/µl)	6.73	3.30-8.60
RBC (10^6/µl)	4.98	3.86-4.92
Hemoglobin (g/dl)	14.6	11.6-14.8
Platelet (10^3/µl)	287	158-348
Total protein (g/dl)	7.7	6.6-8.1
Albumin (g/dl)	4.6	4.1-5.1
Total bilirubin (mg/dl)	0.4	0.4-1.5
AST (U/l)	16	13-30
ALT (U/l)	11	7-23
LDH (U/l)	229	120-245
BUN (mg/dl)	9	8-20
Creatinine (mg/dl)	0.5	0.46-0.79
Sodium (mmol/l)	142	138-145
K (mmol/l)	3.9	3.6-4.8
Cl (mmol/l)	104	101-108
Glucose (mg/dl)	97	73-109
CRP (mg/dl)	0.03	0.00-0.14
APTT (second)	23.9	24.0-39.0
PT (second)	10.5	70-130
D-dimer (µg/ml)	0.5	<1.0
CA125 (U/ml)	99.8	<40
CA19-9 (U/ml)	31	<37
CEA (ng/ml)	1.1	<5
SCC (ng/ml)	1.6	<1.5
HE4 (pmol/l)	27.0	Premenopausal: <70.0; Postmenopausal: <140.0

**Figure 2:**
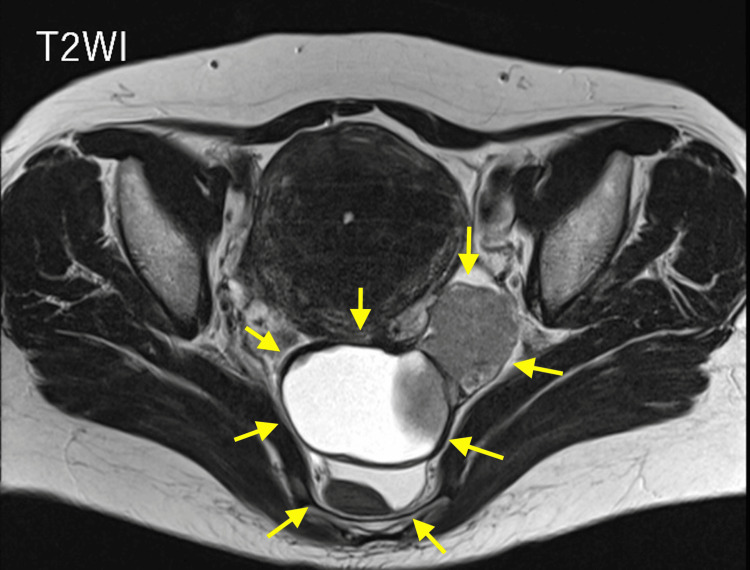
Pelvic contrast-enhanced MRI. The cystic component was considered to be a lesion containing a blood component such as an endometriotic cyst. The solid component was almost homogeneously visualized with a slightly higher signal than the muscle, and the contrast effect was good and homogeneous.

Cervical cytology showed no intraepithelial lesion or malignancy (NILM), and endometrial aspiration histology was negative. Pelvic contrast-enhanced MRI revealed a tumor with both cystic and solid components that appeared to originate from the left ovary with indistinct right adnexa. The cystic component was hyperintense on T1- and T2-weighted images and also hyperintense on fat-suppressed images, suggesting that the lesion contained a blood component such as an endometrioid cyst. The enhancement area was almost homogeneously visualized on T2-weighted images with a slightly higher signal than that of the muscle, and the contrast effect was good and homogeneous. The uterus was enlarged due to myoma and adenomyosis (Figure 2). Diffusion-weighted imaging revealed no significant signal intensity in the tumor region, indicating that the tumor did not exhibit high cellular density.

**Figure 3:**
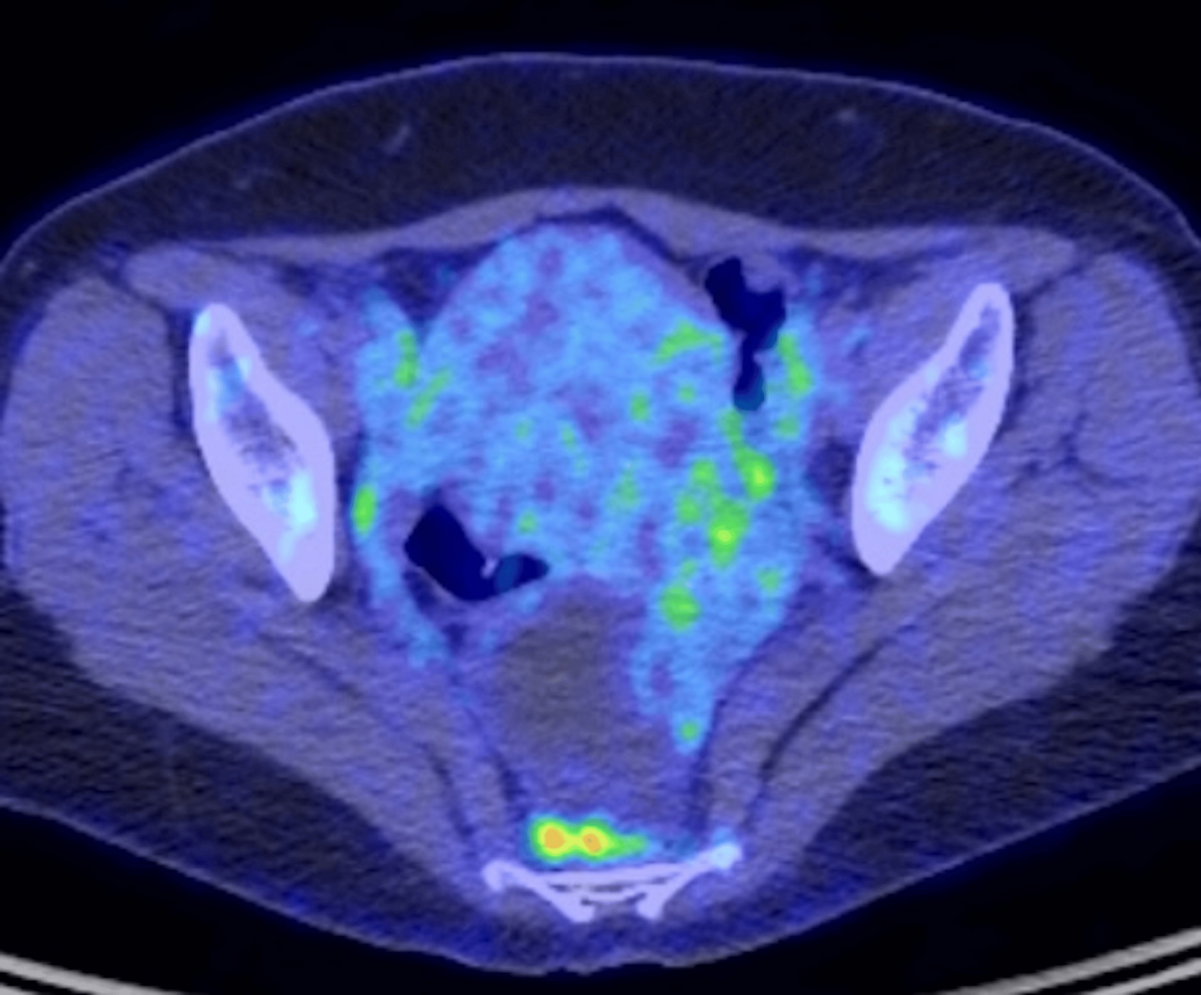
Positron emission tomography-computed tomography (PET-CT) revealed mild enhancement in the left ovarian tumor.

Positron emission tomography-computed tomography (PET-CT) showed a mild accumulation of maximum standardized uptake value (SUVmax) of 3.05 in the left ovarian tumor (Figure 3).

**Figure 4:**
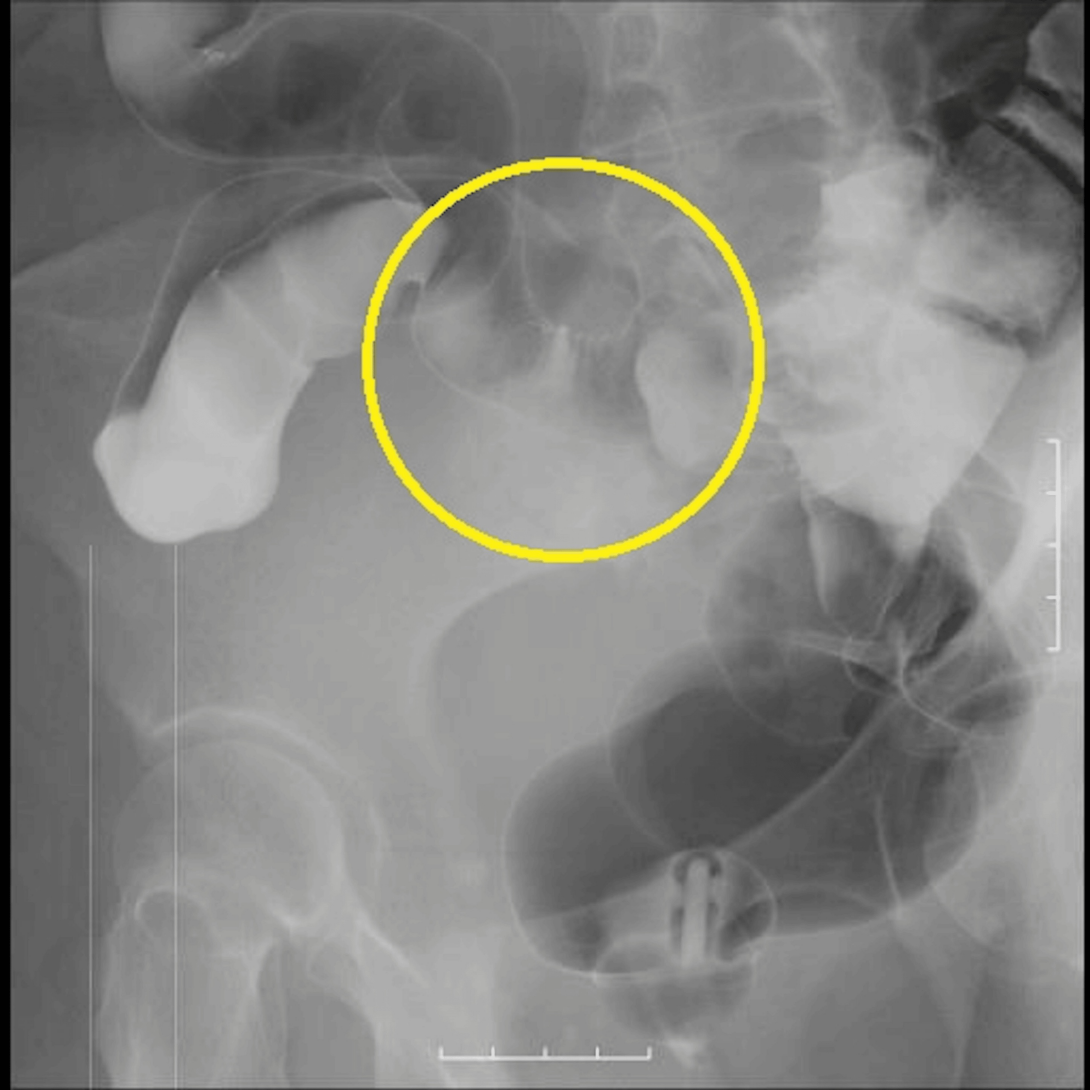
Contrast enema revealed a unilateral spiculated finding in the sigmoid colon, raising suspicion of dissemination.

There were no other findings consistent with distant metastasis. The contrast enema showed a unilateral spiculated finding in the sigmoid colon, which was suspicious of dissemination (Figure 4).

Based on these findings, it was decided to perform primary debulking surgery for preoperative diagnosis of ovarian malignancy. Intra-abdominal findings revealed a small amount of serous ascites fluid, which was submitted for cytological diagnosis. The left ovary was enlarged to 10 cm and adherent to the dorsal surface of the uterus. The omentum showed numerous 1-2 cm disseminated nodules. In addition, a few millimeter-sized disseminated nodule was observed in the mesentery of the small intestine near the ligament of Treitz, a 1 cm nodule at the tip of the appendix and ileocecal region, a few millimeter-sized nodules on the surface of the ileum about 5 cm from the ileocecal region, and a cluster of dissemination less than 1 cm on the surface of the rectum and fat droplets in the rectum. There were no palpable lesions on the abdominal wall, diaphragm, or liver surface.

Intraoperative frozen section diagnosis revealed a granulosa cell tumor on both the left ovary and rectal surface, and since optimal surgery was possible, a simple abdominal hysterectomy, bilateral adnexectomy, subtotal omentectomy, high anterior rectal resection, appendectomy, and disseminated resection were performed.

**Figure 5:**
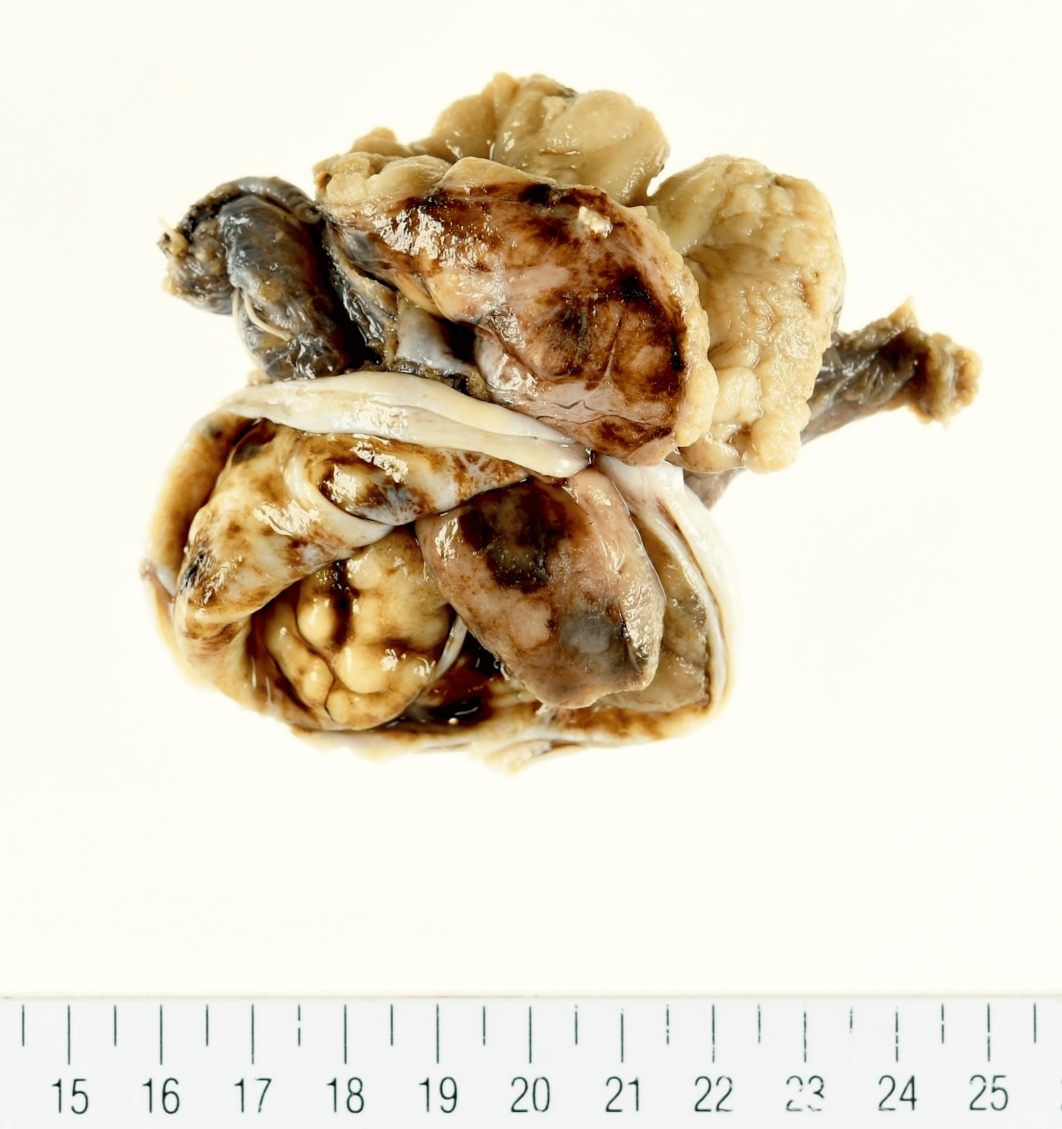
Gross finding. The left ovary was enlarged to 8x6x3 cm and there was a cystic lesion with a solid nodule.

A gross examination of the excised specimen showed that the left ovary was 8x6x3 cm in size with a cystic lesion including a nodule (Figure 5).

**Figure 6:**
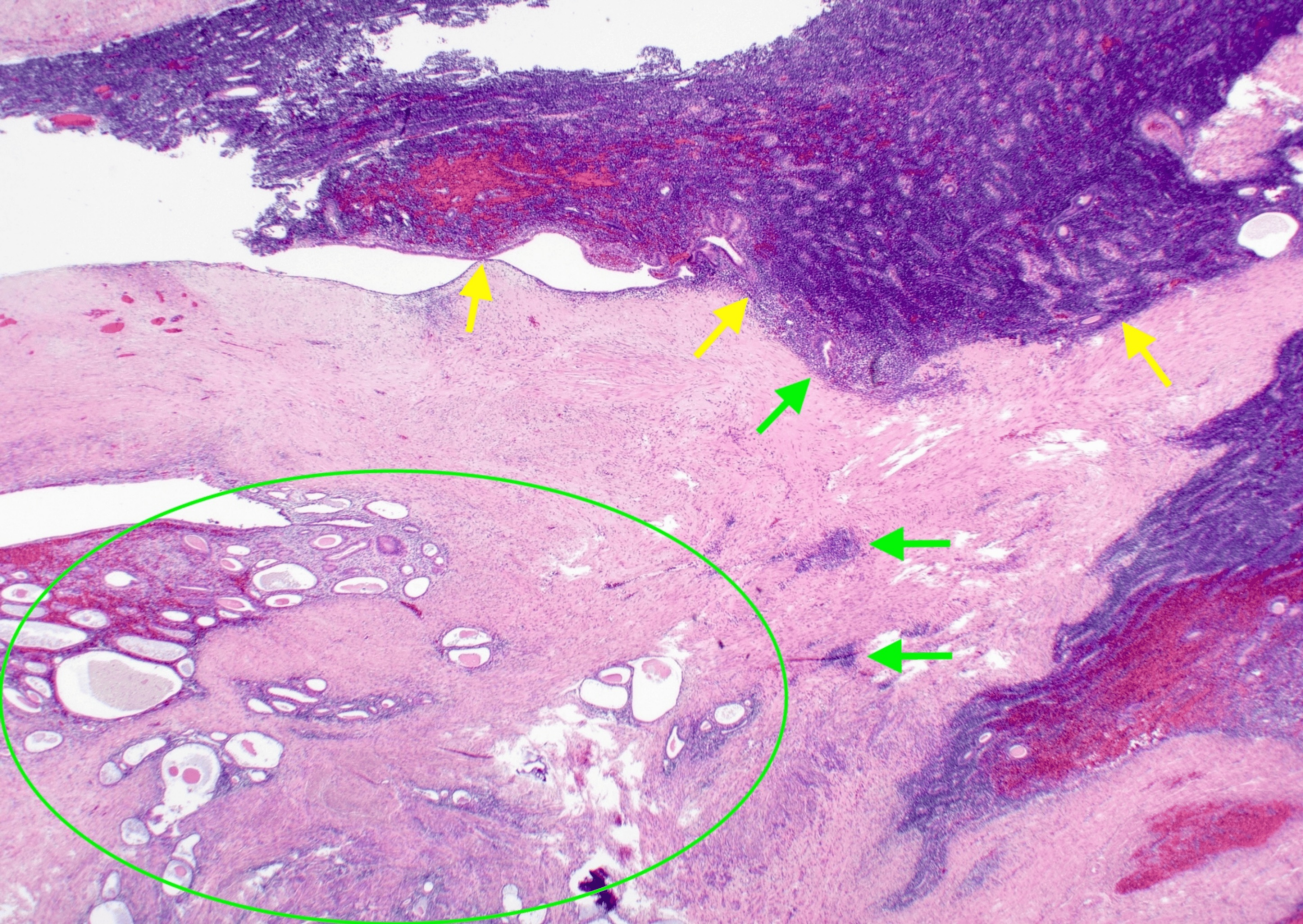
Histologic image. The histologic image shows a tumor resembling proliferative endometrial stroma adjacent to areas of endometriotic lesions. Yellow arrows denote low-grade endometrial stromal sarcoma (LGESS), while green arrows indicate endometrial stroma, and the green circle represents the endometrial gland.

**Figure 7:**
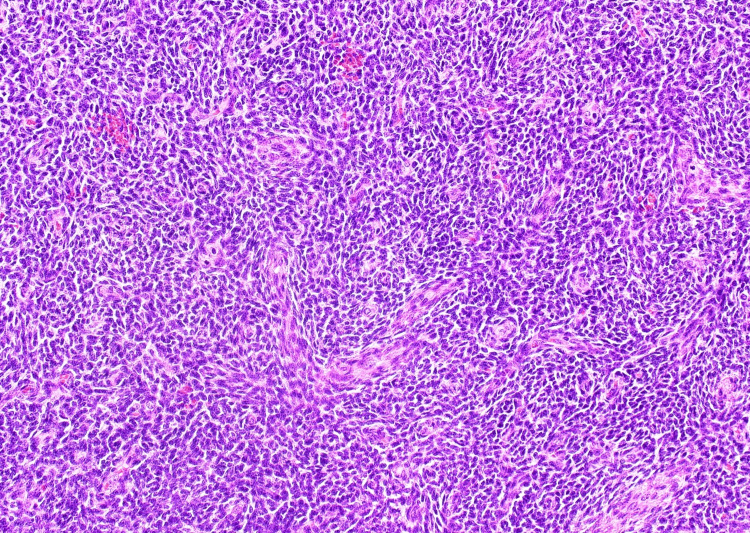
Histologic image. The histologic image shows uniform small to medium-sized tumor cells resembling proliferating endometrial stroma, accompanied by spiral artery-like vessels.

The cavity of the cyst was adherent with clots, and the nodules were white to milky white in color with some hemorrhage. Pathological examination revealed a tumor resembling proliferative endometrial stroma in the left ovary adjacent to an area of endometriotic changes (Figure 6), a longitudinal convoluted proliferation of short spindle-shaped cells with spiral artery-like vessels or flow-like arrangement, or diffuse proliferation without specific structure (Figure 7).

**Figure 8:**
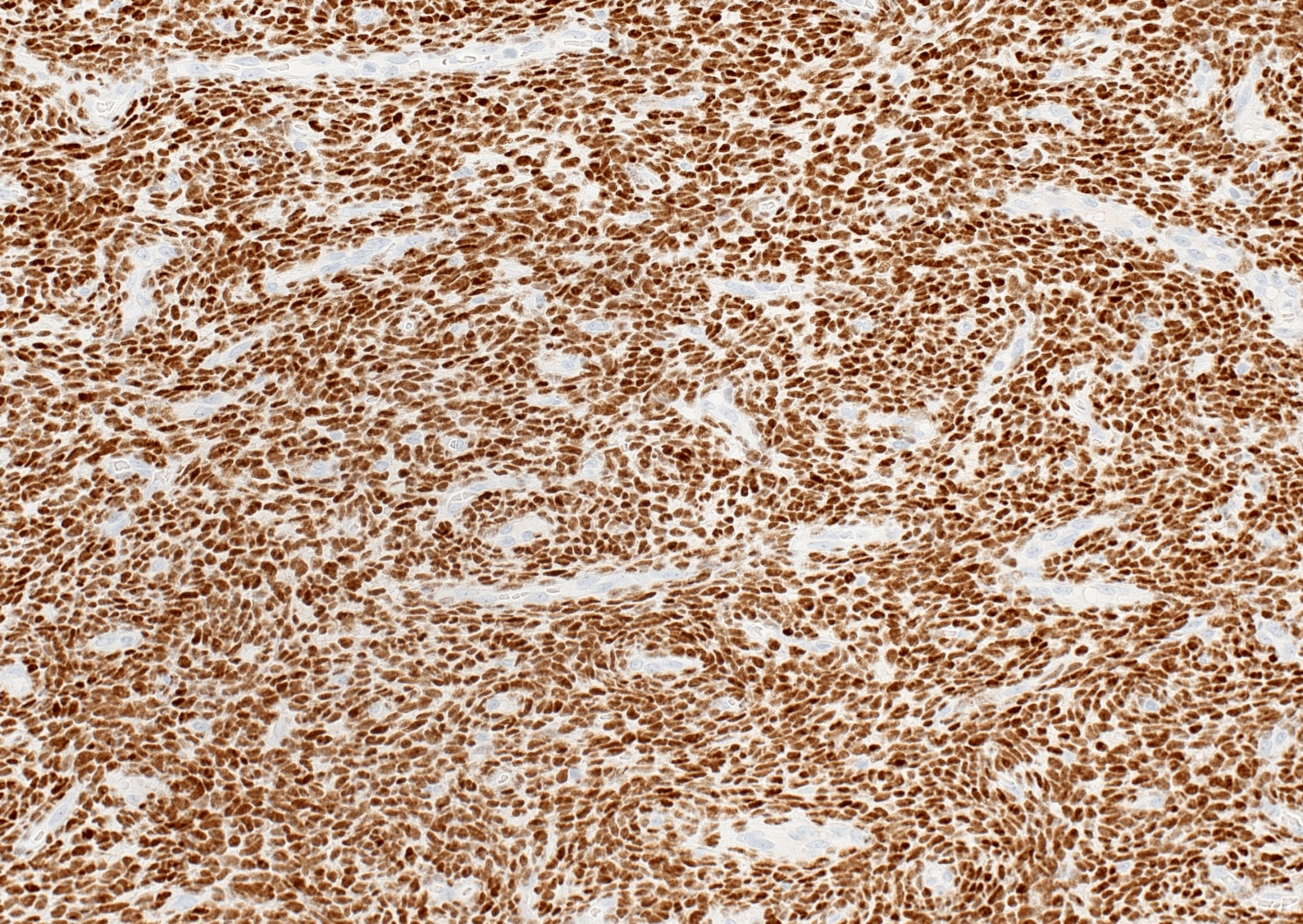
Immunohistochemical finding showed positive staining for the estrogen receptor.

**Figure 9:**
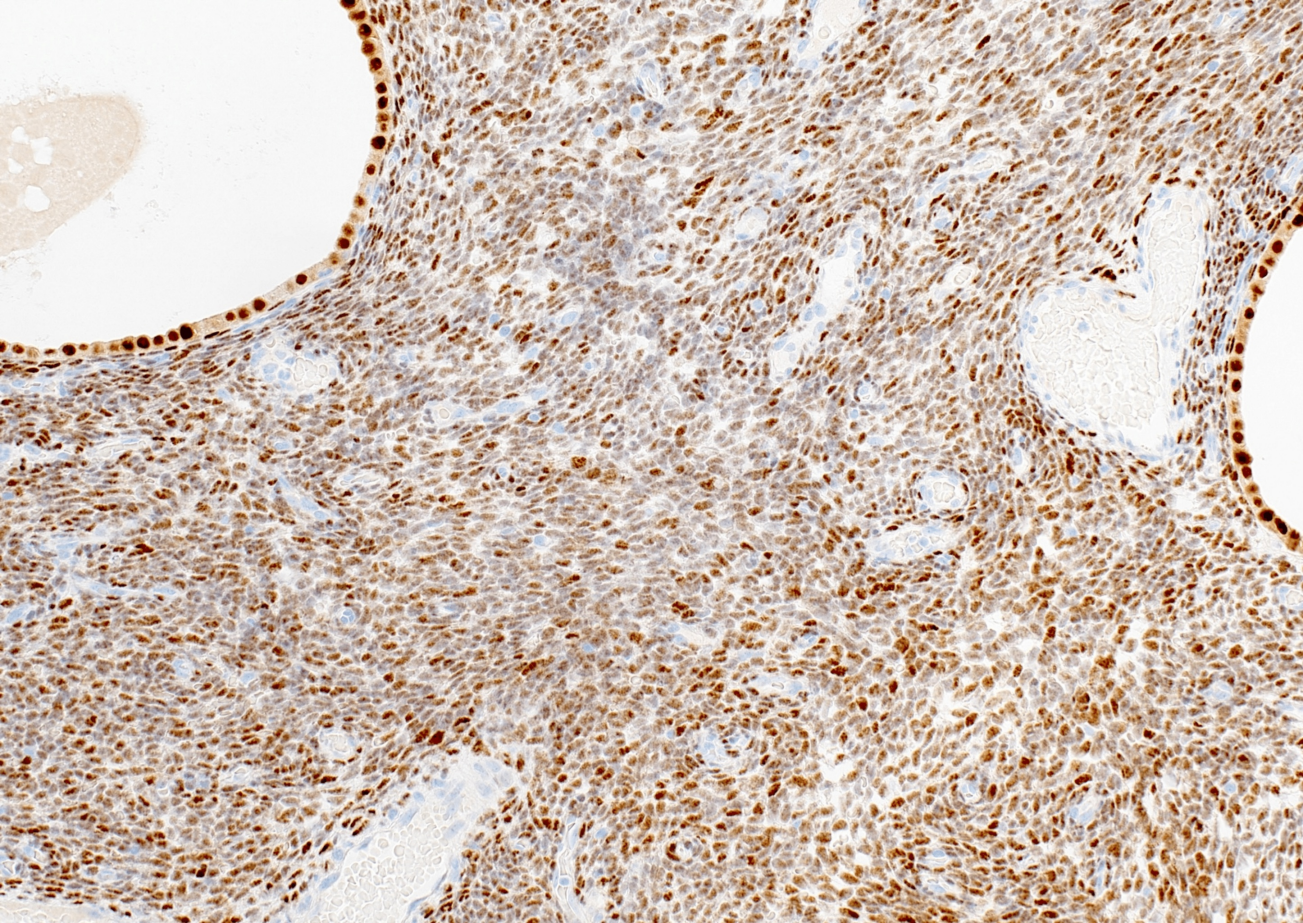
Immunohistochemical finding showed positive staining for the progesterone receptor.

**Figure 10:**
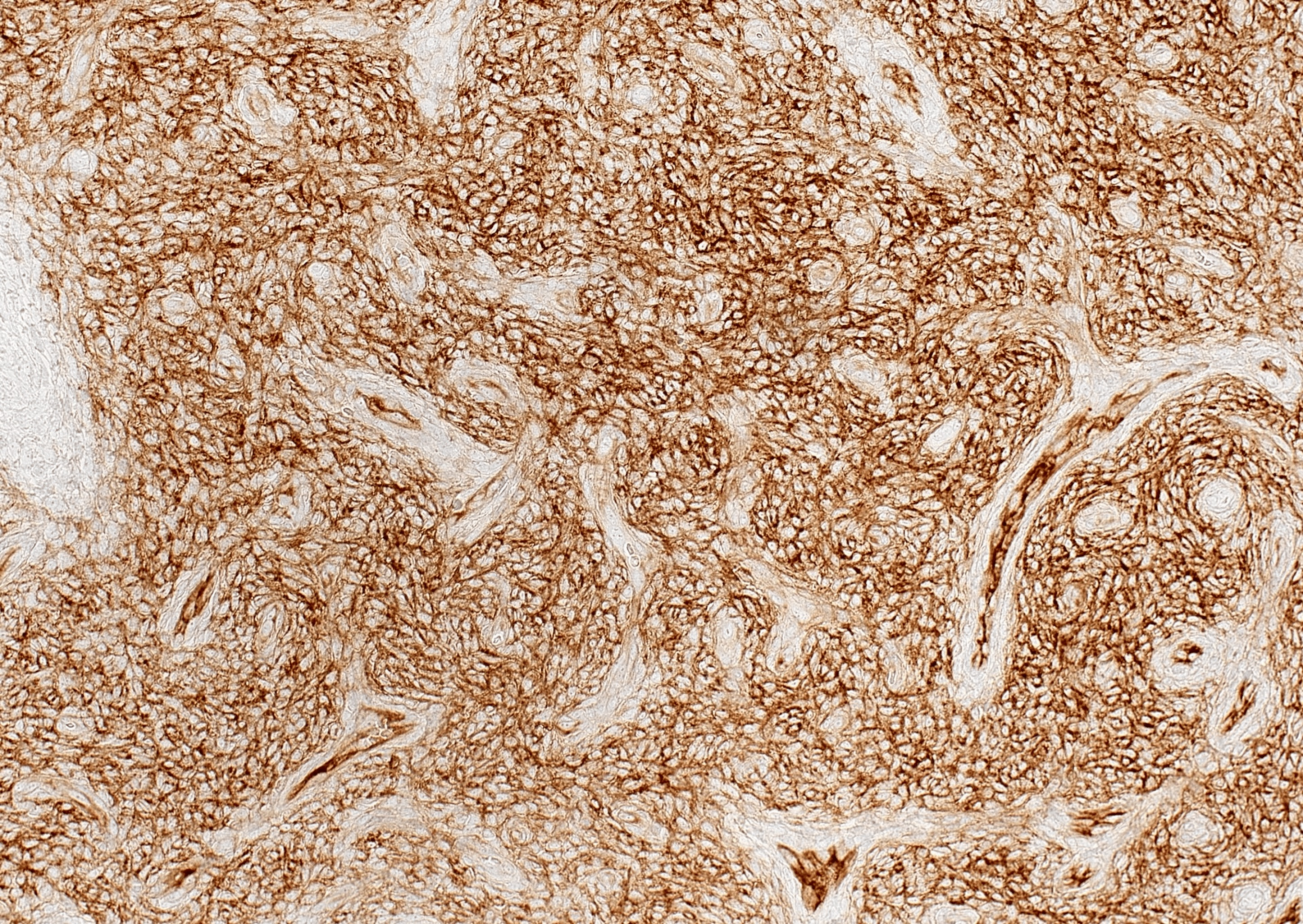
Immunohistochemical finding showed positive staining for CD10.

The boundary between endometriosis stroma and tumor stroma was indistinct. Based on these pathologic findings, the diagnosis of LGESS arising from an ovarian endometrioid cyst was established. Immunohistochemistry showed ER positivity, PgR positivity, CD10 positivity, cyclin D1 negativity, inhibin negativity, and a Ki-67 labeling rate of less than 1% (Figures 8-10).

All disseminated nodules contained ESS and endometriotic lesions were scattered. Invasion into the intrinsic muscularis propria was also observed in the rectum. Vascular invasion was strongly positive and lymphatic invasion was negative. The number of fission images was 0-1/10 high-power fields, and there were no coagulation necrosis images. The uterus exhibited only pathologic findings indicative of uterine myoma and adenomyosis. Ascites cytology was negative. Based on the above pathological findings, this case was finally diagnosed as the International Federation of Gynecology and Obstetrics (FIGO) Stage IIIB (pT3bNxM0).

The patient was started on 600 mg/day of medroxyprogesterone acetate as postoperative therapy one month after surgery. Eight months later, the dose was reduced to 400 mg due to grade 3 weight gain, and the patient continued on the medication. The patient has been free of recurrence for three years after initial treatment.

## Discussion

Primary ovarian LGESS is a rare tumor and is often difficult to distinguish from cases that originate in the endometrium and metastasize to the ovary [5]. Although the mechanism by which it arises from the ovary is still controversial, the presence of endometriosis is strongly suspected to play a role, as 40% of patients with extrauterine LGESS had endometriosis [2]. Recent studies have suggested that the mechanism of tumorigenesis in the context of ovarian endometriotic cysts is that endometriotic tissue forms a chronic inflammatory environment, which may be a promoter of tumorigenesis [6]. In the present case, pathology showed a tumor in continuity with the endometriotic cyst, a finding that supports this hypothesis. At the same time, it suggests the importance of long-term follow-up of patients with ovarian endometriosis. Furthermore, it is likely that excessive estrogen stimulation contributes to tumorigenesis in the progression from endometriotic lesions to LGESS. The fact that ER and PgR were positive in this case also suggests an endocrine-dependent nature of the tumor. ERs and PgRs are positive in 85.4% and 78.0% in LGESS [7]. It has also been suggested that sarcoma-like mural nodules (SLMNs) of ovarian mucinous borderline tumors may induce mesenchymal cell hyperplasia and progress to primary ovarian LGESS [8]; although this is thought to be a very rare case, it may be necessary to keep in mind the possibility that SLMNs may develop via mucinous borderline malignancies in addition to endometriosis.

Extra-uterine ESS is defined by the absence of lesions in the uterus; the diagnosis of primary ovarian LGESS is difficult to make specifically because it is indistinguishable from primary uterine LGESS in terms of clinical presentation [2]. However, primary ovarian LGESS can be diagnosed by symptoms such as renal-ureteric colic due to the relatively early involvement of the surrounding area [9]. On MRI, LGESS is characterized by a high T1 signal, low T2 signal, and heterogeneous post-contrast effects, and the coexistence of findings suggestive of endometriosis, such as the kissing ovaries sign and serosal invasion, may raise the suspicion of primary ovarian LGESS [10]. The diagnosis in this case was finally made by pathology, but the intraoperative frozen diagnosis was considered to be a granulosa cell tumor. Molecular biological analysis has shown that genetic rearrangement of JAZF1-SUZ12 is relatively common in LGESS [11] and has even been detected in cases of primary ovarian ESS [10], so it may be a useful evaluation method when the diagnosis is difficult to make by pathological evaluation alone.

In the previously reported case of primary ovarian LGESS, tumor size, presence of dissemination, and degree of vascular invasion were associated with prognosis. In the present case, although the tumor size was relatively large at 10 cm, it is noteworthy that optimal surgery was achieved and no recurrence was observed for three years. Aggressive resection was also performed for the disseminated lesions observed intraoperatively, and complete resection may have contributed to the improved prognosis. Another noteworthy finding is that lymphatic invasion was negative despite the strong positivity for vascular invasion. This pattern of local tumor spread is similar to that seen in previously reported cases and is a notable feature of LGESS.

Patients effectively benefit from hormonal treatment with aromatase inhibitors [10,12] and gonadotropin-releasing hormone (GnRH) [13] due to the expression of hormone receptors, ER and PgR. Hormonal therapy with medroxyprogesterone acetate, the postoperative treatment of choice in this case, is also an effective treatment modality for ER/PgR-positive LGESS, and in the present case, treatment was started in the first month after surgery and good progress was achieved. On the other hand, weight gain and other adverse effects may be a problem with long-term hormone therapy. In this case, grade 3 weight gain was also observed, requiring a reduction in drug dosage. In this regard, the balance in a long-term treatment strategy should be carefully considered.

## Conclusions

Primary ovarian LGESS is a very rare disease, and the prognosis depends on preoperative suspicion, appropriate choice of surgical procedure, and additional postoperative treatment. In the present case, LGESS arising on the background of ovarian endometriotic cysts was treated with a combination of optimal debulking surgery and hormonal therapy on the background of ER/PgR positivity, and the patient remained recurrence-free for three years postoperatively, which is of clinical significance. The importance of long-term follow-up of patients with ovarian endometriosis and therapeutic management in terms of hormonal therapy for primary ovarian LGESS was reiterated.
